# Effect of biogas sparging on the performance of bio-hydrogen reactor over a long-term operation

**DOI:** 10.1371/journal.pone.0171248

**Published:** 2017-02-16

**Authors:** Chatchawin Nualsri, Prawit Kongjan, Alissara Reungsang, Tsuyoshi Imai

**Affiliations:** 1 Department of Biotechnology, Faculty of Technology, Khon Kaen University, Khon Kaen, Thailand; 2 Faculty of Food and Agricultural Technology, Phibulsongkram Rajabhat University, Pitsanulok, Thailand; 3 Chemistry Division, Department of Science, Faculty of Science and Technology, Prince of Songkla University, Muang, Pattani, Thailand; 4 Bio-Mass Conversion to Energy and Chemicals (Bio-MEC) Research Unit, Department of Science, Faculty of Science and Technology, Prince of Songkla University, Muang, Pattani, Thailand; 5 Research Group for Development of Microbial Hydrogen Production Process from Biomass, Khon Kaen University, Khon Kaen, Thailand; 6 Division of Environmental Science and Engineering, Graduate School of Science and Engineering, Yamaguchi University, Yamaguchi, Japan; National Renewable Energy Laboratory, UNITED STATES

## Abstract

This study aimed to enhance hydrogen production from sugarcane syrup by biogas sparging. Two-stage continuous stirred tank reactor (CSTR) and upflow anaerobic sludge blanket (UASB) reactor were used to produce hydrogen and methane, respectively. Biogas produced from the UASB was used to sparge into the CSTR. Results indicated that sparging with biogas increased the hydrogen production rate (HPR) by 35% (from 17.1 to 23.1 L/L.d) resulted from a reduction in the hydrogen partial pressure. A fluctuation of HPR was observed during a long term monitoring because CO_2_ in the sparging gas and carbon source in the feedstock were consumed by *Enterobacter* sp. to produce succinic acid without hydrogen production. Mixed gas released from the CSTR after the sparging can be considered as bio-hythane (H_2_+CH_4_). In addition, a continuous sparging biogas into CSTR release a partial pressure in the headspace of the methane reactor. In consequent, the methane production rate is increased.

## Introduction

Rising energy demands and concerns about greenhouse gases have increased interest toward hydrogen as a renewable energy carrier due to its high energy content (121 kJ/g), high energy conversion efficiency to electricity and clean combustion [1]. There are several industrial methods for hydrogen production including coal gasification and electrolysis of water, both which consume fossil fuel and electricity as energy sources. An alternative way to produce hydrogen in an environmentally friendly manner is a biological hydrogen production by dark fermentation due to its high production rate, easy and economical operation, and practical at an industrial scale [2].

Several factors influence dark fermentation processes including pH, temperature, substrate type, substrate concentration, inoculum type, hydraulic retention time (HRT), and hydrogen partial pressure. Among these factors, hydrogen partial pressure can cause a serious negative effect on hydrogen production process [3]. High accumulation of hydrogen in the reactor can cause high hydrogen partial pressure in the fermentation broth. Hydrogenase, a key enzyme involving in oxidation and reduction of ferredoxin in hydrogen fermentation process, is directly affected by hydrogen partial pressure. At high hydrogen partial pressure, the enzyme reaction is tended to be reduction of ferredoxin rather than oxidation [4], which decrease the hydrogen production. Moreover, high hydrogen partial pressure could result in metabolic pathways shift toward solventogenesis rather than hydrogen production pathway [5]. Several studies reported the methods to reduce hydrogen partial pressure including agitation [6], enlarging the headspace volume [7], continuous gas releasing [8], and gas sparging.

Gas sparging is recognized as an effective method to reduce hydrogen partial pressure [9]. Commercial gases such as N_2_ [3,10] and CO_2_ [11] as well as CH_4_ [12] obtained from partially purification of biogas were used for sparging to reduce hydrogen partial pressure. Liu et al. [12] used methane sparging to improve hydrogen production. Household solid waste (HSW) was used as substrate to produce hydrogen and methane by two-stage fermentation process. In the first stage, hydrogen was produced from HSW by dark fermentation process. The effluent from hydrogenic reactor was further used in the second step to produce methane by anaerobic digestion process. The methane gas was obtained from purified biogas of methanogenic reactor and used to sparge the hydrogenic reactor. It was found that methane sparging could improve hydrogen production by 88%. Kim et al. [10] studied the effect of gas sparging on continuous hydrogen production by comparison of N_2_ and CO_2_ sparging. Results showed that CO_2_ sparging was more effective than N_2_ sparging. CO_2_ had little effects on hydrogen producers but had inhibitory effect on competitive microorganisms such as acetogens and lactic acid bacteria. These previous reports used the commercial gases to reduce hydrogen partial pressure but the use of commercial gases will increase the operational cost at large-scale applications. Therefore, an approach in this study was to sparge with a low cost gas i.e. biogas from the methanogenic reactor. Since biogas can be collected on-site and directly used for sparging without purification, thus this approach can be economical and practical. In addition, a mixture of gas after biogas sparging can be considered as the bio-hythane (H_2_+CH_4_) which is another beneficial of this approach.

In the present study, the effect of biogas sparging into the hydrogen reactor on the performance of direct integration of two-stage reactors for hydrogen and methane production was evaluated during a long-term operation. The effects of biogas sparging on microbial community in the hydrogen reactor was also investigated.

## Materials and methods

### Feedstock

Sugarcane syrup was used as feedstock for hydrogen fermentation. The method of preparing sugarcane syrup was previously described by Pattra et al. [13]. Sugarcane syrup was diluted with distilled water to a concentration of 25 g COD (chemical oxygen demand)/L and supplemented with inorganic nutrients consisting of (all in mg/L): K_2_HPO_4_ 125, MgCl_2_.6H_2_O 15, FeSO_4_.7H_2_O 25, CuSO_4_.5H_2_O 5, CoCl_2_.5H_2_O 0.125, NH_4_HCO_3_ 5240 and NaHCO_3_ 6720 (modified from Endo et al. [14]). The feedstock was kept in a storage tank at 4°C until it was fed into the reactor.

### Inocula preparation

*Clostridium butyricum* TISTR1032 was used as an inoculum for hydrogen production. The activation and enrichment methods of *C*. *butyricum* followed the method described by Pattra et al. [13]. Anaerobic granular sludge for methane production was collected from a UASB reactor of brewery wastewater treatment plant in Khon Kaen, Thailand. Total solids (TS) and volatile solids (VS) of the granular sludge were 77.6 and 60.1 g/L, respectively. It was kept at 4°C prior the usage.

### Reactor setup and operation

Two-stage hydrogen and methane production was carried out in respective CSTR and UASB reactors with working volumes of 1 L and 24 L, respectively. The CSTR was made from glass with a diameter of 10 cm and a height of 22 cm. It was equipped with a thermometer and a pH probe connected to a digital pH meter (pH 190 Series, Eutech Instruments). Temperature was controlled by a water jacket surrounding the reactor. The water jacket circulated water from water bath (Julabo TW20, Germany) to keep the temperature of the CSTR at 37°C. The reactor was continuously stirred at 150 rpm using a motorized stirrer (Heidon BL1200, SHINTO Scientific, Japan). The UASB reactor was made from acrylic with a diameter of 14 cm and a height of 175 cm. It was operated at room temperature (30±2°C).

**Fig 1:**
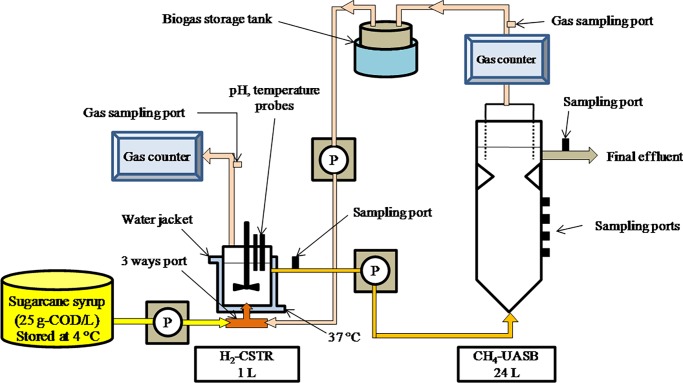
Schematic diagram of the direct integration of two-stage reactor used in this study with biogas sparging system (not subjected to scale).

The CSTR and UASB reactors were directly integrated without buffer tank [15]. The sugarcane syrup at 25 g COD/L was fed into the CSTR to produce hydrogen. Subsequently, hydrogenic effluent from the CSTR was directly fed into the UASB reactor to produce methane. The reactors were operated over 200 days to achieve the optimum HRTs in which the optimal HRT was 3 h in the CSTR and 3 d in the UASB. Upflow velocity of the UASB reactor at HRT of 3 d was 0.02 m/h. Biogas produced at the optimum HRT of 3 d was stored in a storage tank before being pumped into the bottom of the CSTR by a peristaltic pump (Longer Pump^TM^, Model BT100-2J, Taiwan ROC) at a flow rate of 60 mL/min. This flow rate is the average biogas production rate (BPR) of the UASB to disperse the biogas into the CSTR. During the biogas sparging, the CSTR was operated at the HRT of 3 h. The direct integration of two-stage reactor with biogas sparging system is schematically shown in Fig 1. Biogas produced from the UASB and CSTR was measured using a wetted-gas counter. Fermentation broth was collected from the CSTR every 3 days to analyze for COD, total sugar concentration, and organic acids including acetic, butyric, propionic, formic, citric, lactic, and succinic acids.

### Analytical methods

TS and VS of the granular sludge were analyzed according to standard methods [16]. Biogas samples were taken daily from the gas sampling port of the CSTR. The hydrogen, methane and carbon dioxide contents of the biogas were determined using a gas chromatography (GC) (GC-2014, Shimadzu Co. Ltd.) equipped with a thermal conductivity detector (TCD) and a 2-m stainless steel column packed with Shin carbon (50/80 mesh). The GC protocol followed the method of Pattra et al. [17]. The hydrogen and methane volumes in the collected biogas were determined by multiplying the biogas volume by % content of hydrogen or methane. The hydrogen production rate (HPR), and methane production rate (MPR) were expressed as L H_2_/L_reactor_.d, and L CH_4_/L_reactor_.d, respectively. Hydrogen yield (HY) was expressed in mL H_2_/g COD_added_. The measured volumes of hydrogen and methane were expressed at standard temperature and pressure (STP, 0°C and 760 mmHg).

For energy production rate (EPR) calculation, HPR and MPR from two-stage fermentation process in L/L.d units were converted to EPR in units of kJ/L.d by multiplying HPR or MPR with an energy content of 10.8 kJ/L (STP) for hydrogen (equivalent to 121 kJ/g H_2_) or 36 kJ/L (STP) for methane (equivalent to 50 kJ/g CH_4_) [15].

A volume of 2 mL of the fermentation broth from CSTR was centrifuged at 10,000 rpm for 5 min (WiseSpin^®^ CF-10). A volume of 1 mL of supernatant was collected. It was kept at -20°C prior to COD determination by standard methods [16] and total sugar concentration by a phenol sulfuric method using glucose as a standard [18]. Another 0.8 mL of supernatant was acidified by mixing with 0.2 mL of 0.2 M oxalic acid, and filtering through a 0.45 mm cellulose acetate membrane. It was kept at -20°C prior to analysis by high performance liquid chromatography (HPLC) (Shimadzu LC-10AD) for organic acid concentrations. The HPLC was equipped with a VertiSep^TM^ OA 8μm column and a refractive index detector (RID). The temperature of the column was 40°C. H_2_SO_4_ at a concentration of 4 mM was used as the mobile phase at a flow rate of 0.5 mL/min.

To confirm whether the experimental data were consistent with the substrate distribution in continuous hydrogen production, a COD distribution was conducted using the soluble COD values of the products formed (i.e., hydrogen, organic acids, and residual sugars). COD of hydrogen (g COD) was calculated by firstly converting hydrogen production in L unit at STP into mol unit by dividing by 22.4 L. Then, COD of hydrogen (g COD) was calculated by multiplying mol of hydrogen with COD equivalent of hydrogen (16 g COD/mol H_2_).

For acetic, butyric, propionic, formic, lactic, citric, and succinic acids, the COD of each product (g COD) were calculated by multiplying mol of each product with COD equivalent of acetic acid (64 g COD/mol), butyric acid (160 g COD/mol), propionic acid (112 g COD/mol), formic acid (16 g COD/mol), lactic acid (96 g COD/mol), citric acid (144 g COD/mol), and succinic acid (112 g COD/mol), respectively. The COD distribution was calculated using Eq (1).

$$\mathrm{COD}\;\mathrm{distribution}\;\left(\%\right)=\sum \left(\frac{\text{COD product}}{\text{COD initial}}\text{x}100\right)\text{-}100$$ (1)

### Microbial community analysis

Small amounts of the fermentation broth were taken from the sampling ports of the CSTR at Day 15, 30, 45, 60, 90, 110, 140, and 170. The samples were centrifuged at 10,000 rpm for 5 min. The supernatant was discarded. The solids were kept in 50% sterile glycerol at -20°C prior to analyzing its microbial communities using polymerase chain reaction-denaturing gradient gel electrophoresis (PCR-DGGE) following the method of Kongjan et al. [19]. Most of the bands were excised from the gel and re-amplified with primer 357f without a GC clamp or the reverse primer 518r. After re-amplification, PCR products were purified and sequenced by Macrogen Inc. (Seoul, Korea). Closest matches for partial 16S rRNA gene sequences were identified by database searches in GenBank using BLAST [20].

## Results and discussion

### Effects of biogas sparging on the performance of the CSTR

**Fig 2:**
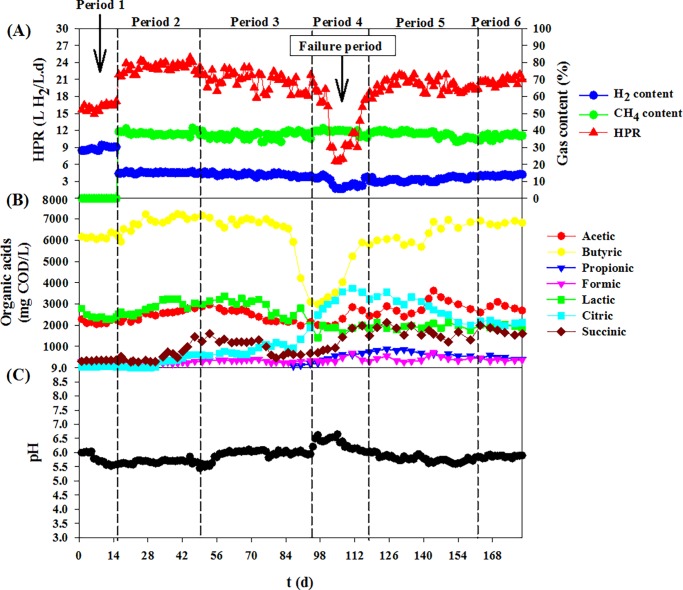
Time course profiles of the continuous stirred tank reactor with biogas sparging during long term monitorin. (A) Hydrogen production rate (HPR), (B) pH, (C) organic acid concentrations.

The two-stage reactor was operated at its optimum HRTs without biogas sparging into the CSTR at the first 15 days of operation (Fig 2A (period 1)). The two-stage reactor showed stable hydrogen and methane production. Average HPR and MPR were 17.1 and 2.1 L/L.d, respectively (Table 1). Hydrogen content in the CSTR of 30.8% was obtained. The average BPR from the UASB was 86.5 L/d with methane content of 64.1%.

**Table 1:** Average hydrogen production rate (HPR), hydrogen yield (HY), hydrogen and methane contents, methane production rate (MPR), and energy production rate (EPR) under steady state conditions (HPR ± ≤10%) at each period during long term monitoring.

Conditions	Period	Days	CSTR					UASB			Total EPR (kJ/L.d)
			HPR (L/L.d)	HY (mL/g COD_added_)	H_2_ (%)	CH_4_ (%)	EPR (kJ/L.d)	MPR (L/L.d)	CH_4_ (%)	EPR (kJ/L.d)	
Non sparging	1	1–15	17.1±0.5	85.5±2.8	30.8±0.8	ND	184.7	2.1±0.1	64.1±1.2	75.6	260.3
With sparging	2	16–50	23.1±0.8	115.4±4.2	15.1±0.4	38.3±1.2	249.5	2.0±0.5	63.0±1.6	72.0	321.5
	3	51–95	20.1±2.1	100.6±10.6	13.7±1.2	37.1±1.8	217.1	2.3±0.6	65.8±3.2	82.8	299.9
	4	96–115	8.3±1.9	41.4±9.5	6.8±1.5	40.1±1.9	89.6	2.4±0.5	70.6±3.4	86.4	176.0
	5	116–162	16.3±2.2	81.5±11.0	11.2±1.2	37.3±2.1	176.0	2.3±0.7	65.1±2.3	82.8	258.8
	6	163–180	20.4±0.5	102.2±2.4	13.3±0.3	36.9±0.9	220.3	2.2±0.2	63.2±1.3	79.2	299.5

ND: Not detected.

CSTR: Continuous stirred tank reactor.

UASB: Upflow anaerobic sludge blanket reactor.

After the first period, the CSTR was sparged with biogas produced from the UASB. The results show that the average HPR after sparging increased from 17.1 L/L.d to 23.1 L/L.d. Hydrogen content in the biogas decreased from 30.8% to 15.1% (Table 1). A decrease in hydrogen content was due to the dilution by the sparging gas. The CSTR showed stable hydrogen production for 35 days after sparging (Fig 2A (period 2)). However, HPR fluctuation was observed from period 3 onwards (51–180 days) (Fig 2A). During period 3 (51–95 days), the HPR was varied within ±10.6% with an average HPR of 20.1 L/L.d and a hydrogen content of 13.7% (Table 1). The decrease in average HPR is corresponded to an increase in succinic acid concentration during period 3–6 (Fig 2B) (see Section “Organic acids”).

During period 4 (96–115 days), a sharp decline in HPR was observed (Fig 2A) due to the contamination of the feedstock by bacteria. The HPR was decreased to an average value of 8.3 L/L.d and hydrogen content was decreased to 6.8%. After cleaning the feedstock tank and replacing with new feedstock, the system was recovered and subsequently monitored until Day 180. It was found that the system could resume to produce hydrogen with an average value of 16.3 L/L.d and hydrogen content of 11.2% in period 5. However, hydrogen production in this period was not stable with the HPR was varied within ±13.5%. During 163–180 days (period 6), the hydrogen production was stable. The system could resume to an average HPR of 20.4 L/L.d and hydrogen content was 13.3%, which was close to the average value observed in period 3 (before a failure period) (Table 1).

Similar trend of HY to HPR was found in every period (Table 1). Maximal HPR and HY of 23.1 L/L.d and 115.4 mL/g COD_added_, respectively, were obtained during the first 35 days with biogas sparging (period 2). Results revealed that sparging with biogas could improve HPR and HY by 35% in comparison to the non-sparging fermentation.

The beneficial of the biogas sparging system is its product i.e., bio-hythane (H_2_+CH_4_) in the CSTR. Unfortunately, the bio-hythane obtained from this study could not be considered yet as a proper gaseous fuel for internal combustion engines. In general, hythane mainly consists of 75–90% (v/v) methane and 10–25% (v/v) hydrogen [21]. However, the maximum hydrogen and methane content obtained from period 2 were respective 15.1 and 38.3% (Table 1) with the rest was CO_2_. This indicates the hydrogen content in the bio-hythane from this study was in a good proportion but methane content was too low, resulted in a low energy content of the bio-hythane. Therefore, in order to raise methane content, the biogas upgrading system such as chemical absorption, water scrubbing, membrane separation, etc. used to remove CO_2_ from the biogas before sparging into the CSTR should be installed. Moreover, CO_2_ in biogas stream released from the CSTR should be removed in order to increase the proportion of hydrogen and methane. If CO_2_ is completely removed from biogas emitted from the CSTR, the final composition of bio-hythane will be 21% hydrogen and 79% methane which falls in a proper compositions of bio-hythane.

### Organic acids

Organic acids detected in the fermentation broth during 180 days of operation including acetic, butyric, propionic, formic, lactic, citric and succinic acids (Table 2). Butyric acid was found as the main component of the hydrogenic effluent. An average butyric acid concentration of 6.17 g COD/L was found at period 1. Butyric acid was increased from 6.17 to 6.83 g COD/L when the CSTR was sparged with biogas (Fig 2B (period 2)). The increase in butyric acid was corresponded to the increase in HPR and HY. This correlation can be illustrated according to the theoretical reaction of butyrate type fermentation (Eq (2)).

C6H12O6→CH3CH2CH2COOH-+2H2+2CO2(ΔG0'=-254.0kJ/mol) (2)

**Table 2:** Organic acids and residual sugars concentrations during long term monitoring of hydrogen production with biogas sparging.

Conditions	Period	Days	Organic acids concentrations (g COD/L)							Residual sugars (g COD/L)	Total COD (g COD/L)
			Acetic	Butyric	Propionic	Formic	Lactic	Citric	Succinic		
No sparging	1	1–15	2.16±0.09	6.17±0.11	ND*	0.19±0.05	2.42±0.16	0.04±0.01	0.33±0.02	9.70±0.15	21.01±0.19
With sparging	2	16–50	2.51±0.22	6.83±0.38	ND*	0.19±0.05	2.84±0.28	0.44±0.14	0.35±0.09	6.78±0.05	19.94±0.10
	3	51–95	2.43±0.29	6.77±0.29	ND*	0.31±0.05	2.88±0.37	0.96±0.33	1.28±0.13	5.92±0.26	20.55±0.32
	4	96–115	2.26±0.37	3.39±0.41	0.49±0.17	0.39±0.14	1.81±0.21	3.18±0.46	1.23±0.53	9.29±0.43	22.04±0.48
	5	116–162	2.86±0.38	6.27±0.45	0.69±0.12	0.43±0.13	1.94±0.12	2.48±0.48	1.65±0.27	4.70±0.24	21.02±0.31
	6	163–180	2.83±0.18	6.82±0.09	0.46±0.07	0.37±0.05	1.96±0.06	2.11±0.09	1.73±0.17	4.45±0.12	20.73±0.18

*ND: Not detected.

From Eq (2), COD equivalent of 1 mol butyric acid and 2 mol hydrogen are 160 g COD and 32 g COD, respectively (equal to butyric/hydrogen ratio of 5:1). Thus, for 1 g COD of butyric acid, 0.2 g COD of hydrogen is produced. Since one mol of hydrogen is equivalent to 16 g COD or 22.4 L at STP, therefore 1 g COD of hydrogen is equal to 1.4 L. Hence, 0.2 g COD of hydrogen is equal to 0.28 L. Our results indicated that butyric acid concentration of 6.83 g COD/L was produced in period 2; hence, only 1.37 g COD of hydrogen should be expected (equal to 1.91 L H_2_/L_substrate_).

$$\begin{array}{l} C_{6}H_{12}O_{6}+2H_{2}O\to 2CH_{3}COOH^{\text{-}}+4H_{2}+2CO_{2}\\ \;\left(\Delta G^{0'}=\text{-}206.0\;kJ/mol\right) \end{array}$$ (3)

For acetate type fermentation, one mol of glucose produces four mol of hydrogen if 2 mol of acetic acid is only by-product (Eq (3)). COD equivalent of 2 mol acetic acid and 4 mol hydrogen are 128 g COD and 64 g COD, respectively (equal to acetic/hydrogen ratio of 2:1). Thus, for 1 g COD of acetic acid, 0.5 g COD of hydrogen is produced (equal to 0.7 L H_2_ at STP). We found that acetic acid concentration of 2.51 g COD/L was produced in period 2 (Table 2); hence, 1.26 g COD of hydrogen was expected (equal to 1.76 L H_2_/L_substrate_).

Therefore, based on the calculation, the estimated total hydrogen produced from butyrate (1.37 g COD of hydrogen) and acetate (1.26 g COD of hydrogen) type fermentation would be 2.63 g COD of hydrogen.

However, 115.4 mL/g COD_added_ of HY was produced in period 2 (Table 1). Since the concentration of substrate was 25 g COD/L, thus the HY of 115.4 mL/g COD_added_ is equal to 2.89 L H_2_/L_substrate_ (equal to 2.06 g COD of hydrogen). The differences between the actual hydrogen produced (2.06 g COD of hydrogen) and the estimated total hydrogen produced (2.63 g COD of hydrogen) from butyrate and acetate type fermentation would be 0.57 g COD of hydrogen. Therefore, the main hydrogen fermentation pathway in this study were acetate and butyrate type fermentation. The differences of 0.57 g COD of hydrogen might be consumed by homoacetogenic bacteria to produce acetic acid.

At period 3 (51–95 days), butyric acid slightly decreased from 6.83 to 6.77 g COD/L while a concentration of succinic acid was sharply increased from 0.35 (period 2) to 1.28 (period 3) g COD/L. An increase in succinic acid concentration was found from period 3 onwards (51–180 days). This could be due to CO_2_ in the biogas might be used to produce succinic acid via succinic acid production pathway (Eq (4)) by *Enterobacter* sp. (see discussions in Section “Effects of biogas sparging on the microbial community in CSTR”).

7C6H12O6+6CO2→12C4H6O4+6H2O(ΔG0'=-119.28kJ/mol) (4)

From Eq (4), succinic acid production is a fermentation pathway that CO_2_ is consumed to produce succinic acid when sugars are used as substrate. Sparging with biogas could increase CO_2_ concentration in the fermentation broth in CSTR since CO_2_ is easily dissolved in water with a much higher solubility than methane [22]. CO_2_ is required to sustain redox and carbon balances in this reaction [23]. Overall conversion of glucose and CO_2_ into succinic acid is thermodynamically favorable with a Gibbs free energy of -119.28 kJ/mol, indicating that succinic acid formation could be occurred easily. Thus, it is confirmed that sucrose in the feedstock and CO_2_ in the biogas were consumed to produce succinic acid without hydrogen formation, resulting in a fluctuation of the HPR during period 3. Moreover, succinic acid in the fermentation broth was observed until period 6 at the concentrations in the range of 1.23–1.73 g COD/L. The results confirmed that CO_2_ in the sparging gas and carbon sources in the feedstock were consumed to produce succinic acid over a long term period.

At period 4 (failure period), butyric acid concentration was dramatically decreased from an average value of 6.77 to 3.39 g COD/L and then increased to approximately 6.27 and 6.82 g COD/L in periods 5 and 6, respectively (Table 2). Correlation between butyric acid concentration and HPR was obviously observed (Fig 1A and 1B). This is not surprising since it is known that hydrogen fermentation pathway of *Clostridium* sp. is butyrate type fermentation [9,13,24]. Propionic acid was firstly found in period 4 (failure period) and remained in the fermentation broth until period 6 (Fig 2B). HPR was sharply decreased during period 4 which could be explained by the fact that sucrose in the feedstock as well as hydrogen were consumed to produce propionic acid as shown in Eq (5).

C6H12O6+2H2→2C3H6O2+2H2O(ΔG0'=-279.4kJ/mol) (5)

Though propionic acid was still found remaining in periods 5 and 6, the hydrogen reactor could maintain its stability to produce hydrogen over these periods. This is because *Clostridium* sp., main hydrogen producer, was resumed to be a predominant specie during period 5 and 6 (see discussions in Section “Effects of biogas sparging on the microbial community in CSTR”).

The pH profile in the fermentation broth during periods 1–6 is shown in Fig 2C. The pH was varied within 5.5–6.0 at every period except at period 4 which the pH was increased to nearly 7.0 (Fig 2C). This was a result of a decrease in the concentrations of acetic, butyric, and lactic acids. Actually, sparging with biogas into the CSTR could decrease pH of the fermentation broth since CO_2_ has a high solubility in water (1.45 g/L of water at 25°C, 100 kPa) in form of carbonic acid (H_2_CO_3_). However, pH in the fermentation broth was not lower than 5.5 which was in the range of optimum pH for hydrogen production. This is because of the buffer capacity of NaHCO_3_ and NH_4_HCO_3_ that were added into the fermentation medium.

### Effects of biogas sparging on the microbial community in CSTR

**Fig 3:**
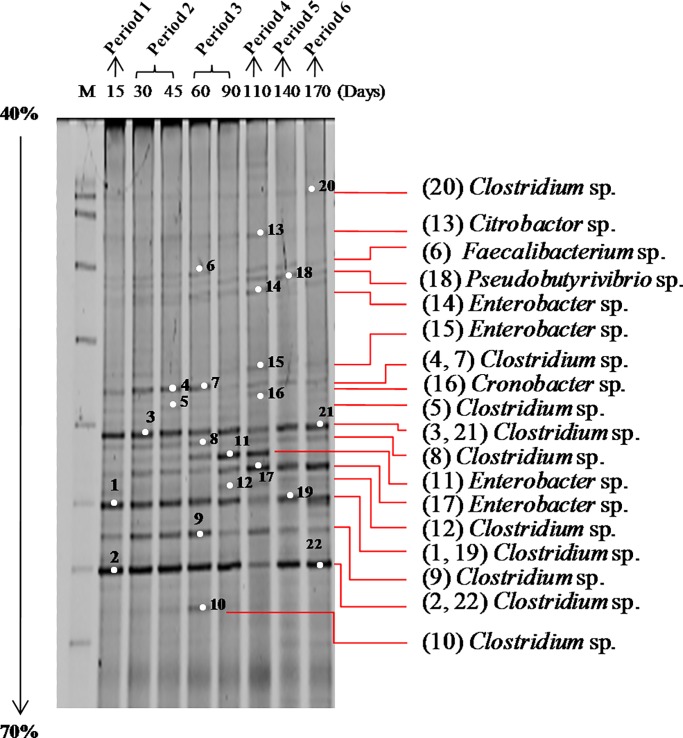
PCR-DGGE analysis of the microbial community in the CSTR with biogas sparging during long term monitoring. M: DGGE marker.

Fig 3 shows the PCR-DGGE analysis of the microbial community in the CSTR during operation with biogas sparging. *Clostridium* sp. was the only predominant species during the first period (band 1, 2). After sparging with the biogas, period 2, *Clostridium* sp. was still a predominant species. However, *Enterobacter* sp. was firstly observed at this period. Further sparging with the biogas until period 3 (51–95 days) revealed that the predominant species were *Clostridium* sp. and *Enterobacter* sp. (band 11). The intensities of both bands were high implying that these bacteria were present at high levels in the reactor [25]. Tanisho et al. [26] reported that *Enterobacter* sp. produced succinic acid in the presence of high levels of CO_2_ in fermentation broths. The increase in *Enterobacter* sp. in the reactor is correlated to an increase in concentration of succinic acid in the fermentation broth during period 3–6 (as mentioned in Section “Organic acids”). During the failure period (days 96–115), *Clostridium* sp. was found at low levels (indicated by light band density) while *Enterobacter* sp. became the predominant species (band 17). After cleaning the feedstock tank and replacing with new feedstock, *Clostridium* sp. resumed as the predominant microorganisms in the fermentation broth which due to the operating conditions of the CSTR were optimized for *Clostridium* sp. [15]. When *Clostridium* sp. was resumed to be the predominant specie during periods 5 and 6, the HPR was increased to 16.3 and 20.4 L/L.d, respectively (as described in Section “Effects of biogas sparging on the performance of the CSTR”). These results suggested that *Clostridium* sp. could maintain their ability to produce hydrogen over long term fermentation.

### Effects of biogas sparging on the performance of the UASB reactor

The performance of the UASB reactor was evaluated during the long term operation by continuous feeding with the hydrogenic effluent from the CSTR to produce methane. Biogas produced from the UASB reactor was continuously released and kept in a storage tank before being sparged into the CSTR. Methane production from the UASB reactor at period 1–6 is shown in Table 1. Generally, methanogenic archaea specifically utilize acetic acid or H_2_/CO_2_ for their growth and producing methane. Other organic acids are to be oxidized into acetic acid and H_2_/CO_2_ in acetogenic stage by obligated proton reducing bacteria under low hydrogen partial pressure [27]. Since hydrogen is mainly generated from fermentation of carbohydrates in the first stage, methanogenic limiting step due to high hydrogen partial pressure could be potentially avoided. Furthermore, continuous and constant removal of biogas from the head space of the second reactor could ensure low accumulation of hydrogen partial pressure [28]. Consequently, an average MPR was evidentially increased from 2.1 L/L.d without biogas sparging to 2.3 L/L.d with biogas sparging from period 3 onward. The increase in MPR during the sparging CSTR with biogas could confirm that other organic acids in the hydrogenic effluent (Table 2) were oxidized effectively to acetate and H_2_/CO_2_ under low hydrogen partial pressure manipulated by continuous removal of biogas from the head space of UASB. Spirito et al. [23] reported that the conversion of the short chain carboxylates into methane by acetogens and methanogens could occur within well-operated anaerobic digestion system.

### COD distribution

The COD distribution for continuous hydrogen production in the CSTR with biogas sparging during a long term monitoring is shown in Table 3. The small errors of COD value were in the ranges of 8.68–12.85%. It is reported that 10% of the biodegradable organic matter is generally utilized for bacterial growth in an anaerobic fermentation [29]. Thus, the results of the COD distribution suggest that the experimental data is accurate.

**Table 3:** COD distribution in continuous hydrogen production with biogas sparging.

Period	Initial (%)	Organic acid concentrations (%)							Residual sugar (%)	H_2_ (%)	Balance (%)
		Acetic	Butyric	Propionic	Formic	Lactic	Citric	Succinic			
1	100	8.64	24.68	0	0.76	9.69	0.14	1.34	38.79	6.11	-9.85
2	100	10.03	27.32	0	0.77	11.35	1.77	1.39	27.13	8.25	-11.99
3	100	9.72	27.08	0	1.24	11.54	3.86	5.11	23.68	7.18	-10.59
4	100	9.03	13.58	1.98	1.55	7.23	12.72	4.92	33.17	2.96	-12.86
5	100	11.46	25.06	2.77	1.72	7.74	11.52	6.41	18.80	5.82	-8.70
6	100	11.31	27.28	1.85	1.47	7.83	8.45	6.93	17.81	7.29	-9.78

### Energy and economic aspects of the biogas sparging

The major benefit of the two-stage anaerobic digestion process is the production of hydrogen and methane. After mixing these two gases, the mixed gas so called hythane is obtained. Normally, the two-stage process produces hydrogen and methane separately. In order to obtain hythane, gas mixing and upgrading system needs to be installed in order to mix hydrogen and methane and to remove carbon dioxide, respectively [28]. Thus, the costs of energy input to operate mixing-upgrading system should be taken into account. However, in this study, biogas generated from methane reactor was used as sparging gas for simultaneously enhancing hydrogen production in the hydrogenic reactor and thus hydrogen can be mixed with methane. Previous report by Willquist et al. [28] indicated that a sparging with biogas into the first reactor could significantly increase hydrogen productivity without any inhibition of osmolarity. In addition, a biogas sparging system could reduce hydrogen partial pressure and consequently increase hydrogen production (up to 35%), leading to a higher total energy in form of hythane in comparison to the non sparging system (Table 1). The costs of energy to drive the sparging pump in the two-stage process with biogas sparging system can be compensated with the costs of energy to drive gas mixing system in the two-stage process without gas sparging. This is because the equipment used in both gas mixing and gas sparging systems are gas blowers or gas compressors, having the same level of power input. Thus, there is no significantly different in energy consumption. However, total energy output from two-stage fermentation process with gas sparging is significantly higher than the two-stage process without gas sparging.

Energy output and economic assessment of two-stage fermentation process without gas sparging were previously assessed by Leite et al. [30]. They studied the performance and energy aspects of single and two-stage anaerobic digestion of waste activated sludge. Results indicated that two-stage anaerobic digestion showed a higher biogas production rate and sludge degradation compared to the single stage. Biogas production rate of 0.87 and 0.55 L/L.d with methane contents of 35 and 69%, respectively, were obtained from the first and the second reactor of two-stage process, which was equal to a total MPR of 0.68 L/L.d and a total EPR of 24.48 kJ/L.d. Economic assessment indicated that payback period of the two-stage process was 3 years. Our data in Table 1 indicated that a two-stage fermentation process with gas sparging gives a maximum total EPR of 321.5 kJ/L.d (period 2), which was 13 times higher than the study of Leite et al. [30]. Moreover, results from a long term monitoring could indicate a stability in hydrogen and methane production of this system. Therefore, the payback period of our two-stage fermentation process with gas sparging could presumably be shorter than 3 years.

## Conclusions

Sparging with biogas enhanced the HPR and HY by up to 35%. A long term monitoring indicated that sparging hydrogen reactor with biogas caused a fluctuation in the HPR. Succinic acid was produced from CO_2_ in the sparging biogas by *Enterobacter* sp. without hydrogen production. MPR was enhanced in the UASB reactor during continuous sparging H_2_-CSTR with biogas. The bio-hythane with a composition of 15.1% H_2_ and 38.3% CH_4_was the by-product obtained from this system. Results indicated the effectiveness of the biogas sparging on the enhancement and performance of the two-stage hydrogen and methane over a long term operation.
